# Organic Egg Consumption: A Systematic Review of Aspects Related to Human Health

**DOI:** 10.3389/fnut.2022.937959

**Published:** 2022-06-24

**Authors:** Arthur Eumann Mesas, Rubén Fernández-Rodríguez, Vicente Martínez-Vizcaíno, José Francisco López-Gil, Sofía Fernández-Franco, Bruno Bizzozero-Peroni, Miriam Garrido-Miguel

**Affiliations:** ^1^Health and Social Research Center, Universidad de Castilla-La Mancha, Cuenca, Spain; ^2^Postgraduate Program in Public Health, Universidade Estadual de Londrina, Londrina, Brazil; ^3^Department of Nutrition, Faculty of Medicine, Universidad Autónoma de Chile, Talca, Chile; ^4^R&D Department, Grupo Avícola Rujamar, Cuenca, Spain; ^5^Department of Physical Education and Health, Higher Institute of Physical Education, Universidad de la República, Rivera, Uruguay; ^6^Faculty of Nursing, Universidad de Castilla-La Mancha, Albacete, Spain

**Keywords:** chicken eggs, health benefits, systematic review, organic food attributes, dietary pattern

## Abstract

Consumption of organic foods has increased recently, but evidence about their potential health benefits is still limited. This systematic review aims to synthesize the available scientific evidence on the association between organic egg consumption and human health. We searched for peer-reviewed articles on this subject indexed in the MEDLINE, EMBASE, Web of Science and Cochrane Library databases from the inception date to April 13, 2022. This review was based on PRISMA guideline recommendations. Three studies on organic egg consumption in humans were included. After 8 weeks of consuming organic eggs, one randomized crossover trial found that participants had higher serum concentrations of the beta-carotene lutein compared to the period without consuming organic eggs. Moreover, in a cross-sectional study with nationally representative data from Americans over the age of 50, it was found that consumption of organic eggs was associated with lower levels of the inflammatory markers C-reactive protein and cystine C compared with conventional eggs. Finally, in a cohort of children aged 0 to 2 years, no significant association was observed between consuming organic eggs and the risk of eczema. In conclusion, the evidence about the potential benefits of organic egg consumption and human health is promising but still requires further research. A human research agenda is proposed based on laboratory studies pointing out that organic eggs have a more desirable nutritional profile than conventional eggs.

## Introduction

There has been a recent increase in the consumption of organic food (1), particularly in developed countries, which may be due to the consumer's perception of the potential effect of this type of production related to sustainability, animal welfare and, especially, of their perception that organic products are healthier than those produced conventionally (1–5). The first two aspects are regulated by the competent authorities that define objective criteria to be applied with respect to the products (e.g., pesticides authorized at certain levels) and processes (e.g., conditions that ensure the natural behavior of the animals) used (6, 7). On the other hand, the concrete effects of organic foods on health depend on being verified in scientific studies, which are still scarce and provide contradictory results (7, 8). It should also be considered that the inconsistent findings on the health effect of the predominantly organic consumption pattern can be explained because the effects may vary according to each food consumed, although to a small extent (9–11).

Among the specific foods included in worldwide dietary patterns that are organically produced is the chicken egg. Eggs are a complete food, providing proteins of high biological value, unsaturated fatty acids, vitamins and minerals with antioxidant potential, and are also widely consumed worldwide due to their affordable market price (12, 13). The production of organic eggs is regulated in Europe and the United States (US) and requires that hens receive feed from organic vegetables that are not only cage-free but also have an outdoor area to move and behave as freely as they did originally (14–16). It is important to note that although there is no consensus yet, biochemical trials have assessed organic eggs as having lower concentrations of environmental contaminants and higher concentrations of micronutrients desirable for their positive health effects (17–20).

Considering that the emerging knowledge on specific organic foods is still in an initial phase, this systematic review was proposed with the aim of synthesizing the available evidence on the association between organic egg consumption and human health. In addition, comments on the research agenda about the proposed topic are presented.

## Methods

A systematic review of the literature was carried out based on the recommendations of the Preferred Reporting Items for Systematic Review and Meta-Analysis (PRISMA) (21) guidelines. The protocol of this review was registered in the PROSPERO database (registration number: CRD42022328052).

We searched the MEDLINE, EMBASE, Web of Science, and Cochrane Library databases for peer-reviewed articles on the relationship between organic egg consumption and human health issues indexed from the inception date to 13 April 2022. No date or language limits will be established. The search syntax included the terms eggs, organic or ecological and human and possible variations combined by means of Boolean operators appropriate to each base. The detailed search strategy is found in the Supplementary Material. In addition, after copying the syntax in the Google Academic search engine, the first twenty pages were observed in search of studies of interest that had not been found in the main databases. With the same objective, the reference lists of the reviews found on the subject were also examined.

The following inclusion criteria were defined according to the *PICOS* structure: a) population: people of all ages; b) intervention/exposure: consumption of organic or ecological eggs; c) comparison: consumption of non-organic eggs, regardless of the type of production method, or no consumption of eggs; d) outcome: any chemical, physical or psychological parameter related to human health; e) study design: cross-sectional or follow-up observational studies or clinical trials. Exclusion criteria were established for not submitting results of the association of interest, when duplicate reports of the same study were submitted or included an ineligible publication format, such as event abstracts, preprints and literature reviews.

After excluding the duplicate studies identified in the different databases, the titles and abstracts were reviewed to rule out those clearly outside the intended scope. Of the remaining studies, the full text was examined to confirm whether the inclusion criteria were met. The data of interest were extracted from the studies finally included. It was not necessary to contact any author to request data not available in the article. Two independent reviewers (AEM and RF-R) extracted the following information from the studies selected for inclusion: author(s) and year; country, design, follow-up, washout, response rate, characteristics of the population, sample size, context, mean age of the participants, women percentage, exposure variable, outcome measures, and main results. A third coauthor (JFL-G) was consulted when disagreements between the initial reviewers occurred.

For the assessment of the risk of bias of each study, the NIH Quality Assessment Tool for Observational Cohort and Cross-sectional Studies (22) was applied, as well as the Rob2 (23) from the Cochrane for the randomized controlled trials. The NIH Quality Assessment Tool for Observational Cohorts and Cross-Sectional Studies was used to assess the risk of bias in cohort and cross-sectional studies. Accordingly, 14 items determined the risk of systematic bias based on clarity of the research question, participation rate, follow up and drop-outs, power analysis, exposure, and outcome time of measurement. The items are scored as “yes,” “no,” “not reported,” “cannot be determined” or “not applicable.” Depending on this, each study was classified as “poor,” “fair” or “good.” Cochrane's Risk-of-Bias 2 (RoB 2) tool was used to assess the risk of bias of the included RCTs, in which the following domains were assessed: randomization process, deviations from intended interventions, missing outcome data, measurement of the outcome, and selection of the reported results. Then, each of the five domains was graded individually as “low risk of bias,” “some concerns” or “high risk of bias” 0.26 Finally, each study was classified for the overall risk of bias as (1) “low risk of bias” when a low risk of bias was determined for all domains; (2) “some concerns” when at least one domain was assessed as raising some concerns but not to be at high risk of bias for any single domain; or (3) “high risk of bias” when a high risk of bias was reached for at least one domain or some concerns in multiple domains.

The included studies were analyzed separately, and a synthesis of their main characteristics and results was developed.

## Results

**Figure 1:**
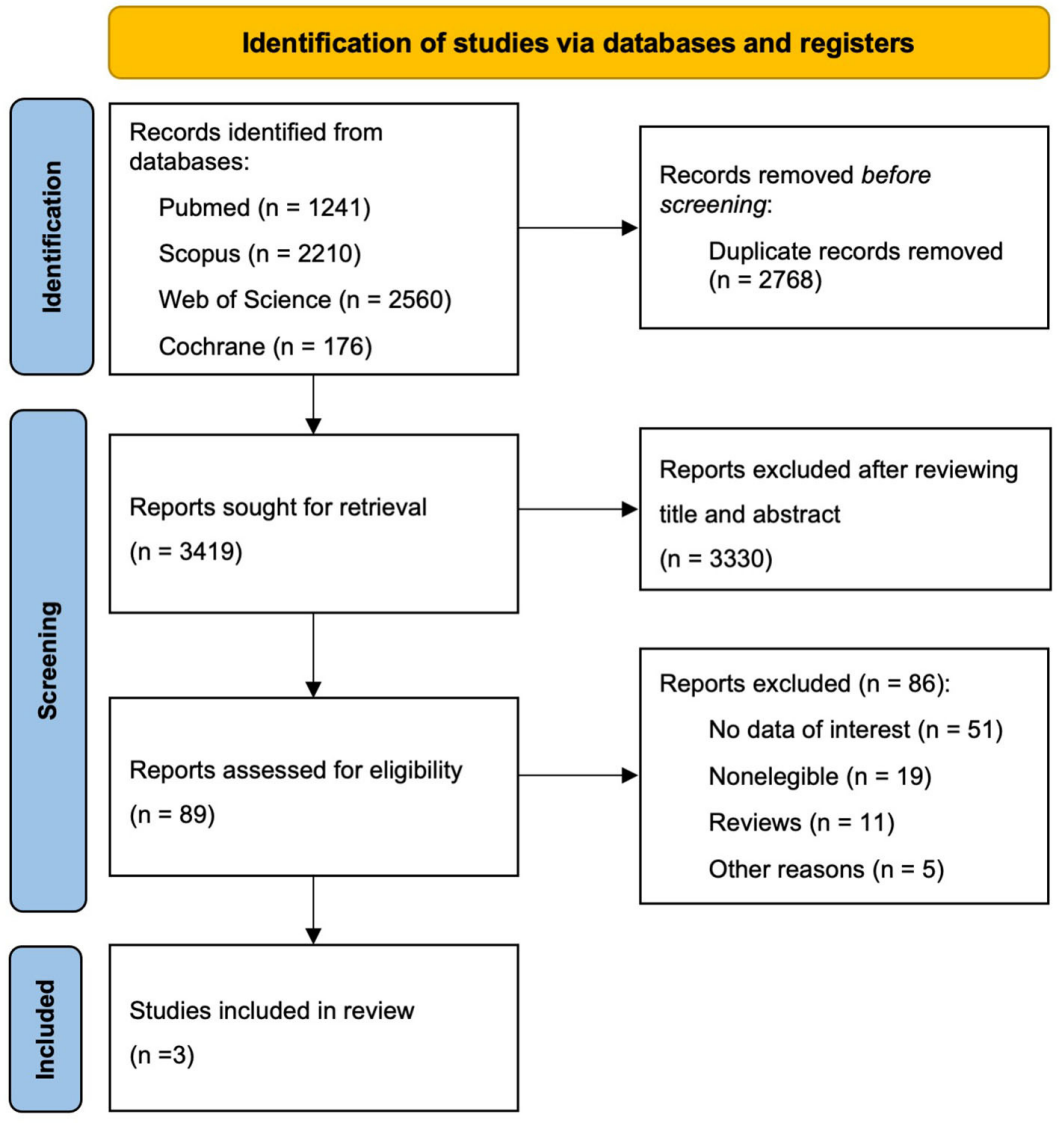
PRISMA 2020 flow diagram of study selection.

Out of a total of 3,419 articles identified in the search, 3330 were discarded due to the title and abstract. Of the remaining 89, reading the full text resulted in the exclusion of 87 studies for the reasons specified in Figure 1. Three studies (10, 11, 24) were finally included in this systematic review.

Table 1 presents the main characteristics and results of the included studies. The oldest study (2008) was conducted in The Netherlands (10), and the next two were conducted in the United States (11, 24). Each study had a different type of design. In the first study, a cohort of 2,583 newborns was followed up to 2 years of age with the aim of analyzing whether the consumption of organic foods, including eggs, moderate (from 50 to 90% of consumption occasions) or strict (more than 90% of the time) was associated with an increased likelihood of developing eczema compared with eating conventional eggs (i.e., less than half of the time choosing organic eggs) (10). Although the crude analyses indicated unfavorable results for the consumption of organic eggs (i.e., greater probability of having eczema), when controlling for the confounding effect of other exposures, such as breast-feeding, pet, day-care, tobacco, etc., that detrimental effect was not sustained.

**Table 1:** Characteristics of the included studies.

**Author, year**	**Study**	**Population**	**Exposure variable**	**Dependent variables**	**Main results**
	**Country** **Design** **Follow-up** **Washout** **Response rate**	**Sample size** **Context** **Mean age** **% Female**	
Burns-Whitmore et al. (24)	United States Randomized crossover trial 8 weeks 4 weeks 76.9%	20 Lacto-ovo-vegetarian adults 38 ± 3 years 80.0%	Six organic eggs/week (intervention) *vs*. no eggs (control)	Serum carotenoids: Lutein ß-carotene Zeaxanthin	Compared with the control, in organic egg treatment lutein was significantly higher (*p* = 0.009), ß-carotene increased only approached significance (*p* = 0.066) and zeaxanthin was not associated (*p* = 0.139).
Kummeling et al. (10)	The Netherlands Cohort NA NA 94.5%	2583 children 2 years 49.0%	Moderate (50–90%) and strictly (>90%) organic egg *vs*. conventional (<50%) egg consumption	Eczema	In analysis adjusted for sociodemographic and several other exposures (breast-feeding, pet, day-care, tobacco, etc.), neither moderate (OR: 1.40; 95% CI: 0.98, 1.99) nor strictly (OR: 1.03; 95% CI: 0.76, 1.38) organic egg consumption was associated with eczema.
Ludwig-Borycz et al. (11)	United States Cross-sectional NA NA 47.3%	3815 Free-living >50 years 63.5 ± 14.6 years 57.3%	Organic eggs *vs*. no organic eggs	C-reactive protein Cystatin C	In analysis adjusted for sociodemographic and lifestyle confounders including caloric intake, organic egg consumers had lower CRP (log: −0.091; 95% CI: −0.181, −0.001) and CysC (log: −0.046; 95% CI: −0.071, – 0.022).

*CI, confidence interval; CRP, C-reactive protein; CysC, Cystatin C; NA, Not applicable; OR, odds ratio*.

The second study, conducted in 2012, was a randomized crossover clinical trial including 20 free-living lacto-ovo-vegetarian adults with the aim of analyzing the transfer of 3 types of carotenoids from eggs to blood serum (24). In the intervention phase, the participants received 6 organic eggs per week for 8 weeks, and with a washout time of 4 weeks, the control phase was carried out, in which they did not consume eggs. The final results revealed an increase in beta-carotene lutein after eating organic eggs compared to the period without eating eggs.

On the other hand, the third study was a cross-sectional design in which 3,815 adults over 50 years of age were questioned about the consumption or non-consumption of organic eggs, and they had blood samples collected to assess the levels of inflammatory markers (11). In the analysis adjusted for sociodemographic and lifestyle confounders, including caloric intake, organic egg consumers had slightly lower levels of C-reactive protein (CRP) and cystatin C (CysC) than those consuming conventional eggs (11) (Table 1).

Finally, the risk of bias assessment of the included studies revealed that among observational studies, one scored as “Good” (10) and the other as “Fair” (11) quality according to the NIH tool. The randomized controlled trial had an overall risk of bias of some concerns (24). The detailed risk of bias assessment of each study is found in the Supplementary material (Table S1 and Figure S1).

## Discussion

This systematic review is pioneering in studying the relationship between organic egg consumption and aspects related to human health. To date, this topic has been explored in only three studies with different designs and population groups studied. Although two of the studies reported favorable results in terms of higher serum carotenoid levels (24) and lower levels of specific inflammatory markers (11) associated with the consumption of organic eggs, this limited set of results does not yet allow firm conclusions to be drawn about the benefits of organic eggs on human health.

Egg allergy is the second most common food allergy in infants and young children after cow's milk allergy and can pose quality of life concerns (25). In addition to avoiding egg consumption, it has been reported that the introduction of eggs in the diet between 3 and 6 months of life could reduce the risk of egg allergy (26). With the expansion of organic agriculture and its possible lower allergenic potential, justified by the higher concentration of nutrients with anti-inflammatory potential such as omega-3 polyunsaturated fatty acids (20), it has been suggested that the intake of organic eggs could also represent a dietary alternative for children allergic or predisposed to egg allergy. Although the KOALA Birth Cohort Study conducted in The Netherlands found no prospective association between moderate or strict consumption of organic eggs (as well as organic meat, fruits, and vegetables) in infants from birth to 2 years of age, the authors reported that the consumption of organic dairy products was associated with a lower risk of eczema (10). In quantitative terms, exposure to egg proteins is expected to be lower than exposure to milk throughout this stage of life. For this reason, the advancement of knowledge about the allergenic potential of organic vs. conventional eggs still depends on longer-term prospective studies with repeated measurements of the consumption of eggs and other organic foods.

In the randomized crossover clinical trial conducted by Burns-Whitmore et al. (24), a higher concentration of lutein in the blood of lacto-ovo-vegetarian adults was observed when they consumed approximately one organic egg daily compared to when they did not consume eggs (24). Elevated levels of this carotenoid in the blood may play an important role in the prevention of macular degeneration and age-related vision loss (27). Although, according to the authors, organic eggs provide a bioavailable source of lutein (a carotenoid with anti-inflammatory properties), it is necessary to consider that the control group did not consume eggs of any kind. For this reason, it could not be affirmed that the contribution of carotenoids was higher when organic eggs were consumed instead of conventional eggs.

Regarding the third and the most recent scientific evidence on the subject, in the nationally representative, longitudinal panel study of Americans over 50, significantly lower levels of two important inflammatory markers were found among individuals who consumed organic eggs compared to those who consumed conventional or non-organic eggs. It is noteworthy that these results were observed independently of several important confounders, such as body mass index, blood pressure, diabetes, physical activity, and total daily caloric intake. Considering the accumulation of evidence on the inflammatory potential of the diet in cardiometabolic risk (28), the aforementioned study sheds light on the possible additive anti-inflammatory effect of organic vs. non-organic feeding (24). However, these findings certainly need to be confirmed in future prospective studies and, furthermore, replicated in populations with dietary patterns different from the Westernized, predominant in the country studied, the United States.

Our results indicate that scientific evidence has thus far not focused on whether organic eggs are directly associated with health benefits. What has indeed advanced—not only for eggs but also for other organically produced foods—was research on the nutritional value of organic foods compared to conventional foods (6), which in turn could lead to advantages for human health (7). For instance, organic vegetables have fewer environmental pollutants (29), which are related to cardiovascular risk (30) and cancer (31). In addition, organic foods of animal origin provide a better lipid profile, vitamins and minerals involved in the etiology of many diseases throughout the life cycle, as well as lower levels of contamination by microorganisms related to gastrointestinal disorders (7, 32).

Specifically, it has been found in studies based on biochemical analyses of egg composition that, compared to conventional eggs, organic eggs are lower in saturated fat (conventional eggs: 85.7 g kg−1 yolk; organic eggs: 68.1 g kg−1 yolk) (20), have a lower n-6/n-3 ratio (conventional eggs: 11.5; organic eggs: 7.8) (20), and have fewer endocrine disruptors such as dimethyl phthalate (DMP) (conventional eggs: 76%; organic eggs: 52%) (33). In addition, other review studies reported that organic eggs are less exposed to antibiotics (34) and are less contaminated by Salmonella (35). These biological differences are supposed to be largely explained by animal feed based on feed made with mostly organic products and under extremely hygienic conditions (29, 36). In particular, on the diet of the hens in different types of creation, compared to the feed commonly used in conventional cage creation, organic farming (and in cage-free farming) favors the predominant consumption of grass from external areas, which provides high amounts of fiber, tocopherol, carotenoids and flavonoids (20), allowing a remarkable transfer of bioactive substances that could confer higher nutritional quality to the organic egg compared to the conventional egg. In addition, it has also been shown that cage-free housing and the guarantee of adequate outdoor space for the animal behavior of all livestock ensures that laying hens can move freely during some hours of the day and, consequently, are less stressed (37). It is understandable that the result of this set of measures based on sustainability, high-quality animal nutrition, and animal welfare results in a feed that generates a lower environmental footprint (38, 39) and, above all, a higher nutritional density (6) compared to conventional egg production. It is also reasonable to consider that future clinical and epidemiological studies could test the hypothesis of whether these better nutritional qualities translate into concrete benefits for people who consume eggs from organic farms.

The main limitation of this review is the small number and heterogeneous design of the included studies, which limits the conclusions that can be drawn and does not justify recommendations on the consumption of organic eggs. In particular, it is noteworthy that the three studies used different comparison groups to analyze the potential benefits of organic egg consumption, which prevents comparability between them. In the two observational studies, the comparison group was composed of individuals who consumed organic eggs less than 50% of the time (10) or did not consume organic eggs (11), while the experimental study considered not consuming eggs of any type as the comparison group (24). The scarcity and methodological heterogeneity of studies are inherent limitations at the early stage of research on any organic food. The main reasons why no progress has yet been made in understanding whether adherence to a dietary pattern based on organic food translates into benefits for human health are that consumption is still very low and strictly related to socioeconomic status (40) and, consequently, to better health status.

In conclusion, preliminary evidence from human studies suggests that organic eggs may have nutritional advantages over conventional or non-organic eggs, possibly related to the higher levels of carotenoids and the reduction in the inflammatory potential of the diet. We advocate the inclusion of questions about the frequency of consumption of organic foods in future population-based dietary surveys. In addition, on the one hand, it is important to defend that laboratory studies are essential to discover biological differences related to the composition of organic foods and have thus far presented promising results in their favor. Last, knowledge about the real impact of organic agricultural production, and in particular of organic eggs, on human health requires clinical trials with samples, follow-up time and control of several confounding variables specifically designed for that purpose.

## Author Contributions

VM-V, AM, and MG-M conceived the present idea. AM and MG-M performed the literature searches and data extraction. RF-R and BB-P assessed the risk of bias of the included studies. AM drafted the first manuscript version. All authors provided intellectual content and approved the final manuscript version.

## Funding

The present study was funded by Grupo Avícola Rujamar, San Lorenzo de la Parrilla, Spain. The funder had no role in the execution of the search, selection and evaluation of studies, interpretation of data, or decision to present the results.

## Conflict of Interest

The authors declare that the research was conducted in the absence of any commercial or financial relationships that could be construed as a potential conflict of interest.

## Publisher's Note

All claims expressed in this article are solely those of the authors and do not necessarily represent those of their affiliated organizations, or those of the publisher, the editors and the reviewers. Any product that may be evaluated in this article, or claim that may be made by its manufacturer, is not guaranteed or endorsed by the publisher.

## References

[1] Hansmann R Baur I Binder CR. Increasing organic food consumption: an integrating model of drivers and barriers. J Clean Prod. (2020) 275:123058. 10.1016/j.jclepro.2020.123058

[2] Thøgersen J. Country differences in sustainable consumption: the case of organic food. JMK. (2010) 30:171–85. 10.1177/0276146710361926

[3] Goetze F Ferjani A Götze F Ferjani A. Who buys organic foods in Switzerland? Agrarforschung Schweiz. (2014) 5:338–43.

[4] Joo D-N Li C Khan A Qalati S Naz S Rana F. Purchase intention toward organic food among young consumers using theory of planned behavior: role of environmental concerns and environmental awareness. J Environ Plan. Manag. (2020) 64:796–822. 10.1080/09640568.2020.1785404

[5] Ashraf M. What drives and mediates organic food purchase intention: an analysis using bounded rationality theory. J Int Food Agribus. Mark. (2020) 33:1–32. 10.1080/08974438.2020.1770660

[6] Mie A Andersen HR Gunnarsson S Kahl J Kesse-Guyot E Rembiałkowska E. Human health implications of organic food and organic agriculture: a comprehensive review. Environ Health. (2017) 16:111. 10.1186/s12940-017-0315-4 29073935 PMC5658984

[7] Brantsæter AL Ydersbond TA Hoppin JA Haugen M Meltzer HM. Organic food in the diet: exposure and health implications. Annu Rev Public Health. (2017) 38:295–313. 10.1146/annurev-publhealth-031816-044437 27992727

[8] Kamalakannan S Mathieu IP. Organic food: nutritional and environmental considerations. Pediatr Rev. (2021) 42:345–7. 10.1542/pir.2020-002980 34074724

[9] Dangour AD Lock K Hayter A Aikenhead A Allen E Uauy R. Nutrition-Related health effects of organic foods: a systematic review. Am J Clin Nutr. (2010) 92:203–10. 10.3945/ajcn.2010.29269 20463045

[10] Kummeling I Thijs C Huber M van de Vijver LPL Snijders BEP Penders J. Consumption of organic foods and risk of atopic disease during the first 2 years of life in the Netherlands. Br J Nutr. (2008) 99:598–605. 10.1017/S0007114507815844 17761012

[11] Ludwig-Borycz E Guyer HM Aljahdali AA Baylin A. Organic food consumption is associated with inflammatory biomarkers among older adults. Public Health Nutr. (2021) 24:4603–13. 10.1017/S1368980020005236 33353578 PMC10195235

[12] Miranda JM Anton X Redondo-Valbuena C Roca-Saavedra P Rodriguez JA Lamas A. Egg and egg-derived foods: effects on human health and use as functional foods. Nutrients. (2015) 7:706–29. 10.3390/nu7010706 25608941 PMC4303863

[13] Réhault-Godbert S Guyot N Nys Y. The golden egg: nutritional value, bioactivities, and emerging benefits for human health. Nutrients. (2019) 11:684. 10.3390/nu11030684 30909449 PMC6470839

[14] Campmajó G Núñez O. Authentication of Conventional and Organic Eggs. In: Chromatographic and Related Separation Techniques in Food Integrity and Authenticity. Munich: World Scientific Publishing Co Pte Ltd. p. 187–213.

[15] Alagawany M Abd El-Hack ME Farag MR. Nutritional Strategies to Produce Organic and Healthy Poultry Products. Cham: Springer International Publishing (2019). p. 339–56.

[16] United States Department of Agriculture. USDA Organic. Guidelines for Organic Certification of Poultry. (2022). Available online at: https://www.ams.usda.gov/rules-regulations/organic (Accessed April 23, 2022).

[17] Banaszewska D Biesiada-Drzazga B Marciniuk M Hrnčár C Arpášová H Kaim-Mirowski S. Comparison of the quality of cage and organic eggs available in retail and their content of selected macro-elements. Acta Sci Pol Technol Aliment. (2020) 19:159–67. 10.17306/J.AFS.2020.0797 32600012

[18] Marelli SP Madeddu M Mangiagalli MG Cerolini S Zaniboni L. Egg production systems, open space allowance and their effects on physical parameters and fatty acid profile in commercial eggs. Animals. (2021) 11:265. 10.3390/ani11020265 33494383 PMC7911268

[19] Methner U Diller R Reiche R Böhland K Boehland K. Occurence of salmonellae in laying hens in different housing systems and conclusion for the control. Berl Munch Tierarztl Wochenschr. (2006) 119:467–73. 17172134

[20] Mugnai C Sossidou EN Dal Bosco A Ruggeri S Mattioli S Castellini C. The effects of husbandry system on the grass intake and egg nutritive characteristics of laying hens. J Sci Food Agric. (2014) 94:459–67. 10.1002/jsfa.6269 23775487

[21] Page MJ McKenzie JE Bossuyt PM Boutron I Hoffmann TC Mulrow CD. The Prisma 2020 statement: an updated guideline for reporting systematic reviews. BMJ. (2021) 372:n71. 10.1136/bmj.n71 33782057 PMC8005924

[22] National Institutes of Health. Quality Assessment Tool for Observational Cohort and Cross Sectional Studies. (2014). Available online at: https://www.nhlbi.nih.gov/health-pro/guidelines/in-develop/cardiovascular-risk-reduction/tools/cohort (a=ccessed 23 April, 2022).

[23] Sterne JAC Savovic J Page MJ Elbers RG Blencowe NS Boutron I. Rob 2: a revised tool for assessing risk of bias in randomised trials. BMJ. (2019) 366:l4898. 10.1136/bmj.l4898 31462531

[24] Burns-Whitmore BL Haddad EH Sabaté J Jaceldo-Siegl K Tanzman J Rajaram S. Effect of N-3 fatty acid enriched eggs and organic eggs on serum lutein in free-living lacto-ovo vegetarians. Eur J Clin Nutr. (2010) 64:1332–7. 10.1038/ejcn.2010.140 20664616

[25] Caubet JC Wang J. Current understanding of egg allergy. Pediatr Clin North Am. (2011) 58:427–43, xi. Epub 2011/04/02. 10.1016/j.pcl.2011.02.014 21453811 PMC3069662

[26] Al-Saud B Sigurdardottir ST. Early introduction of egg and the development of egg allergy in children: a systematic review and meta-analysis. Int Arch Allergy Immunol. (2018) 177:350–9. 10.1159/000492131 30184525

[27] Nolan JM Stack J O'Connell E Beatty S. The relationships between macular pigment optical density and its constituent carotenoids in diet and serum. Invest Ophthalmol Vis Sci. (2007) 48:571–82. 10.1167/iovs.06-0864 17251452

[28] Hariharan R Odjidja EN Scott D Shivappa N Hebert JR Hodge A. The Dietary inflammatory index, obesity, type 2 diabetes, and cardiovascular risk factors and diseases. Obes Rev. (2022) 23:e13349. 10.1111/obr.13349 34708499

[29] Ramakrishnan B Maddela NR Venkateswarlu K Megharaj M. Organic farming: does it contribute to contaminant-free produce and ensure food safety? Sci Total Environ. (2021) 769:145079. 10.1016/j.scitotenv.2021.145079 33482543

[30] O'Toole TE Conklin DJ Bhatnagar A. Environmental risk factors for heart disease. Rev Environ Health. (2008) 23:167–202. 10.1515/reveh.2008.23.3.167 19119685

[31] Vogt R Bennett D Cassady D Frost J Ritz B Hertz-Picciotto I. Cancer and non-cancer health effects from food contaminant exposures for children and adults in California: a risk assessment. Environ Health. (2012) 11:83. 10.1186/1476-069X-11-83 23140444 PMC3551655

[32] Lairon D. Nutritional quality and safety of organic food. Agron Sustain Dev. (2010) 30:33–41. 10.1051/agro/2009019

[33] Kuzukiran O Yurdakok-Dikmen B Sevin S Sireli UT Iplikcioglu-Cil G Filazi A. Determination of selected endocrine disruptors in organic, free-range, and battery-produced hen eggs and risk assessment. Environ Sci Pollut Res Int. (2018) 25:35376–86. 10.1007/s11356-018-3400-5 30343372

[34] Abd El-Hack ME El-Saadony MT Salem HM El-Tahan AM Soliman MM Youssef GBA. Alternatives to antibiotics for organic poultry production: types, modes of action and impacts on bird's health and production. Poultry Sci. (2022) 101:101696. 10.1016/j.psj.2022.101696 35150942 PMC8844281

[35] Sosnowski M Osek J. Microbiological safety of food of animal origin from organic farms. J Vet Res. (2021) 65:87–92. 10.2478/jvetres-2021-0015 33817400 PMC8009579

[36] Williams CM. Nutritional quality of organic food: shades of grey or shades of green? Proc Nutr Soc. (2002) 61:19–24. 10.1079/pns2001126 12002790

[37] Bryden WL Li X Ruhnke I Zhang D Shini S. Nutrition, feeding and laying hen welfare. Animal Production Sci. (2021) 61:893–914. 10.1071/AN20396

[38] Anderson KE. Overview of natural and organic egg production: looking back to the future1 1papers from the current and future prospects for natural and organic poultry symposium were presented at the poultry science association's 97th annual meeting in Niagara Falls, Ontario, Canada. J Applied Poul Res. (2009) 18:348–54. 10.3382/japr.2008-00119

[39] Cattell Noll L Leach AM Seufert V Galloway JN Atwell B Erisman JW. The nitrogen footprint of organic food in the United States. Environ Res Letters. (2020) 15:045004. 10.1088/1748-9326/ab702925875026

[40] Strzok JL Huffman WE. Willingness to pay for organic food products and organic purity: experimental evidence. AgBioForum. (2015) 18:345–53.

