# Supplementary material for: Organic Egg Consumption: A Systematic Review of Aspects Related to Human Health
Source: Front Nutr. 2022 Jun 24;9:937959. doi: 10.3389/fnut.2022.937959 (PMC9263557; doi:10.3389/fnut.2022.937959)
Supplement: Supplementary file 1 [file Data_Sheet_1.docx]

**SUPPLEMENTARY MATERIAL**

**Search strategy** (April 13, 2022)

- **PUBMED** (N=1241)

egg[Title/abstract] AND (organic[Title/abstract] OR ecologic[Title/abstract])

- **SCOPUS** (N=2210)

ABS (egg) AND ABS (organic OR ecological) AND ABS (children OR adults OR youth OR humans OR elderly)

- **WEB OF SCIENCE** (N=2560)

AB=(egg) AND AB=(organic OR ecological) AND AB=(children OR adults OR youth OR humans OR elderly)

- **COCHRANE LIBRARY** (N=176)

(egg) AND (organic OR ecological) AND (children OR adults OR youth OR humans OR elderly)

**Table S1.** National Institute of Health (NIH) quality assessment for cohort and cross-sectional studies.

| **Criteria** | **Kummeling et al., 2008** | **Ludwig-Borycz et al., 2020** |
| --- | --- | --- |
| 1. Was the research question or objective in this paper clearly stated? | Y | Y |
| 2. Was the study population clearly specified and defined? | Y | Y |
| 3. Was the participation rate of eligible persons at least 50%? | Y | N |
| 4. Were all the subjects selected or recruited from the same or similar populations (including the same time period)? Were inclusion and exclusion criteria for being in the study prespecified and applied uniformly to all participants? | Y | Y |
| 5. Was a sample size justification, power description, or variance and effect estimates provided? | N | N |
| 6. For the analyses in this paper, were the exposure(s) of interest measured prior to the outcome(s) being measured? | Y | Y |
| 7. Was the timeframe sufficient so that one could reasonably expect to see an association between exposure and outcome if it existed? | Y | NA |
| 8. For exposures that can vary in amount or level, did the study examine different levels of the exposure as related to the outcome (e.g., categories of exposure, or exposure measured as continuous variable)? | Y | Y |
| 9. Were the exposure measures (independent variables) clearly defined, valid, reliable, and implemented consistently across all study participants? | Y | Y |
| 10. Was the exposure(s) assessed more than once over time? | N | N |
| 11. Were the outcome measures (dependent variables) clearly defined, valid, reliable, and implemented consistently across all study participants? | Y | Y |
| 12. Were the outcome assessors blinded to the exposure status of participants? | NA | NA |
| 13. Was loss to follow-up after baseline 20% or less? | Y | NA |
| 14. Were key potential confounding variables measured and adjusted statistically for their impact on the relationship between exposure(s) and outcome(s)? | Y | Y |
| **Quality Rating (Good, Fair, or Poor)** | **Good** | **Fair** |

**Y**: yes; **N**: no; **NA**: not applicable.


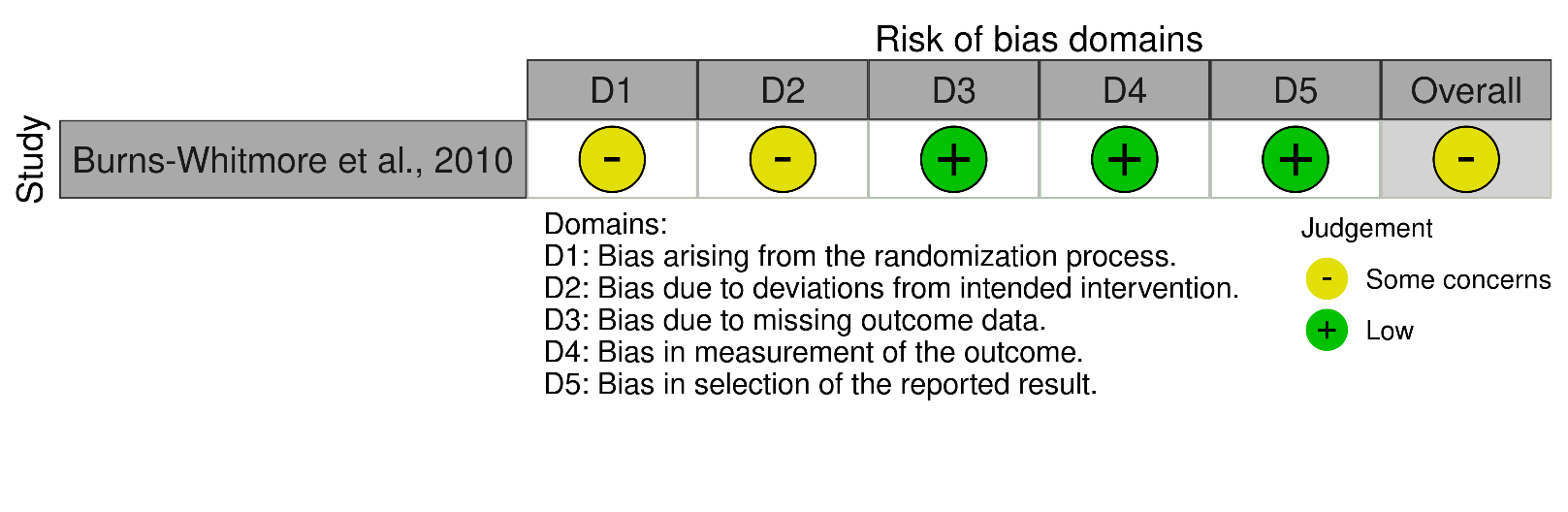


**Figure S1.** Quality assessment for randomized controlled trial (RoB2).
